# Low-cost clamp for the measurement of vegetation spectral signatures

**DOI:** 10.1016/j.ohx.2024.e00557

**Published:** 2024-07-07

**Authors:** Camilo Acevedo-Correa, Manuel Goez, Maria C. Torres-Madronero, Tatiana Rondon

**Affiliations:** a Research Group on Smart Machine and Pattern Recognition, MIRP Laboratory, Instituto Tecnológico Metropolitano, Calle 54 # 31-10, Medellín 050012, Colombia; b Department of Computer and Decision Sciences, Universidad Nacional de Colombia, AV 80 65-223, Medellin 050034, Colombia; c Corporación Colombiana de Investigación Agropecuaria (AGROSAVIA), Centro de Investigación La Selva, Rionegro, Antioquia 250047, Colombia

**Keywords:** Spectral signature, Precision agriculture, Vegetation spectrum, Leaf, Low-cost clamp

## Abstract

Spectral signatures allow the characterization of a surface from the reflected or emitted energy along the electromagnetic spectrum. This type of measurement has several potential applications in precision agriculture. However, capturing the spectral signatures of plants requires specialized instruments, either in the field or the laboratory. The cost of these instruments is high, so their incorporation in crop monitoring tasks is not massive, given the low investment in agricultural technology. This paper presents a low-cost clamp to capture spectral leaf signatures in the laboratory and the field. The clamp can be 3D printed using PLA (polylactic acid); it allows the connection of 2 optical fibers: one for a spectrometer and one for a light source. It is designed for ease of use and holds a leave firmly without causing damage, allowing data to be collected with less disturbance. The article compares signatures captured directly using a fiber and the proposed clamp; noise reduction across the spectrum is achieved with the clamp.

## Graphical abstract

**Figure:**
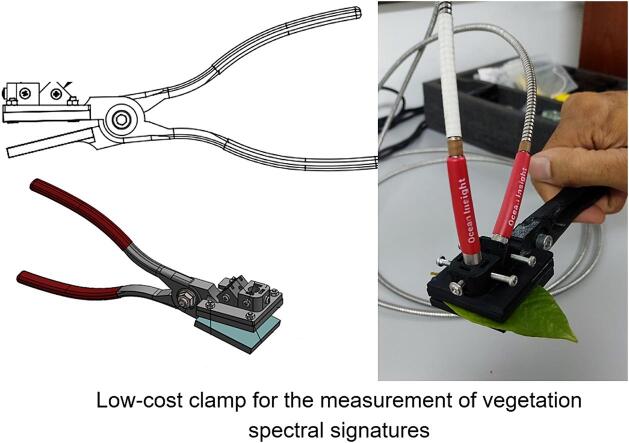

Specifications table.

**Table:** 

Hardware name	Low-cost clamp for leaf spectroscopy
Subject area	● Engineering and materials science Environmental, planetary, and agricultural sciences
Hardware type	● Measuring physical properties and in-lab sensors Field measurements and sensors
Closest commercial analog	Leaf Clip Part# CLP040001 of Spectral Evolution
Open source license	Attribution-ShareAlike 4.0 International (CC BY-SA 4.0)
Cost of hardware	5.16 USD (without additional equipment)
Source file repository	https://doi.org/10.17605/OSF.IO/VN79E

## 1 Hardware in context

In precision agriculture, spectroscopy is recognized as one of the technologies with great potential for crop monitoring [1]. This is due to the possibility of measuring the energy emitted and reflected by a surface along the electromagnetic spectrum using different sensors and systems over remote platforms (e.g., unmanned aircraft, airplanes, satellites) [2] as well as close-range devices (e.g., spectrometers) [3]. Several previous studies show the potential of the spectral signature of vegetation for the monitoring of phenological phenomena [4], [5] and the health conditions of a plant [6]. The spectral signature of a plant has a low reflectance in the visible range, which is associated with pigments such as chlorophyll [1]. Instead, the spectral signature of vegetation has a high reflectance close to the near-infrared related to the health state of the plant. The region where this abrupt change in reflectance occurs is called the red-edge region [1]. Despite several studies showing the potential of spectrometry for crop monitoring [7], [8], [9], [10], these technologies still need to be widely used in commercial crops. The main limitation of their widespread use in practical and commercial applications is the high cost of the devices to measure the spectral signature [11].

Usually, field measurement requires a system that integrates a spectrometer, a light source, fiber optics, and a clamp that allows interaction with the plant leaf. The clamp, the device that helps to fix the leaf, illuminate, and capture the reflectance, is marketed by different companies but requires considerable investment. For instance, Spectral Evolution has a clamp (USD 4,295, including white reflectance and light source) that works integrally with the spectrometer for use in the field, which facilitates the collection of samples of plant leaves in laboratories or crops [12], [13]. The model CI-710LP from CID Bio-Science includes a clamp and a mini spectrometer [14] to capture leaf transmission, reflectance, and absorbance between 400 to 950 nm (USD 7,490, including a spectrometer). The ASD leaf clip (USD 1,626, including white and black standards) requires the accessories ASDA Plant probe (USD 1,724) [15].

This article presents a low-cost clamp design accessible to the agriculture sector, which can be 3D printed using PLA. This design seeks to reduce costs in data capture for precision agriculture applications, but also from the search for acquisition protocols that allow repeatability and reliability of data. The clamp has been used to capture spectral signatures in crops such as maize, as presented in [16], and avocado [17].

## 2 Hardware description

This work introduces an open-source design of a mouth clamp to measure the electromagnetic spectrum of plant leaves. This clamp can be integrated with a spectrometer and a light source. The clamp was constructed to stabilize the variation of the data and the light reflected by the plant when capturing the signatures with the spectrometer. The tool is a 3D-printed gripper made of PLA material and has a handle to make it easy. PLA is the ideal material for manufacturing the gripper due to its ease of printing, low cost, and suitability for a working environment with low climatic conditions, around 20 degrees Celsius. However, PETG can be considered the gripper's manufacturing material in more extreme climatic conditions due to its high heat resistance, impact resistance, and strength.

The clamp has holes to insert two optical fibers: the spectrometer and the light source. Two flat vanes inside the clamp have two points where the optical fiber of the light hits the sample, and the optical fiber of the spectrometer captures its reflection to capture the spectral signatures. This clamp includes three different bodies: duckbill clamp 1.2, duckbill clamp 2.2, and a separate path for the fiber connector, in case you need to add a support or guide component to fix the fibers. The last component was specifically designed for the SMA905 connector, where the fiber optic fibers are assembled using M6 screws.

The clamp is designed to be used in heavy environments due to its simple and comfortable design, providing stability when taking samples in the agricultural field. Being a lightweight and easy-to-assemble piece, it poses no problems for use by any user. The tool made of PLA is resistant to different types of stress, locations, and climates in Colombia. The experiments conducted with the clamp were conducted in the municipality of Rionegro, located in the department of Antioquia, where the average temperature is 16.06 °C and the relative humidity is 84.92 %. Similarly, the clamp was tested in the department of Córdoba, in the municipality of Montería, where the average temperature is 26.77 °C and the relative humidity is 84.42 %. The experiments were conducted from 7:00 h to 16:00 h in Colombia. Both experiments were conducted in the open field, where none of the environmental variables affected the measurements.

Considering the above, the tool will provide the following advantages:

• It facilitates data collection in the field and laboratory.
• It is a light and comfortable element for the user due to its materials, size, and shape.
• Spectral signatures collected with our clamp present few variations during acquisition since the fixation of fibers and the leaf provided by the clamp body.

## 3 Design files summary

**Fig. 1:**
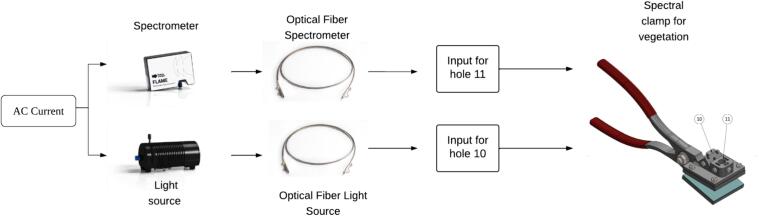
Block diagram of the clamp's connection to the spectrometer.

The block diagram in Fig. 1 provides an example setup illustrating how the clamp can be used to measure plant spectral signatures. The power supply is connected to the spectrometer and the light source, the fiber optic ports, and, in this case, the SMA905 at the corresponding outputs of the light source and the spectrometer. Finally, the other SMA905 ports of the fiber optics are secured to the spectral vegetation clamp at ports 10 and 11.

The design files include:

• The upper part of the clamp with the fiber connector support: Duckbill plier 1.2
• The lower part of the clamp with the locking mechanism: Duckbill plier 2.2
• The fiber optic terminal connection base: Fiber connector support
• The total assembly of the clamp: Ensamblaje plier

## 4 Bill of materials summary

This section presents a list of the components required for the construction of the tool, including possible prices and sources of purchase*.*

If the hex head screw m6 x 16 mm is unavailable, it can be replaced by a different head screw.

## 5 Build instructions

**Fig. 2:**
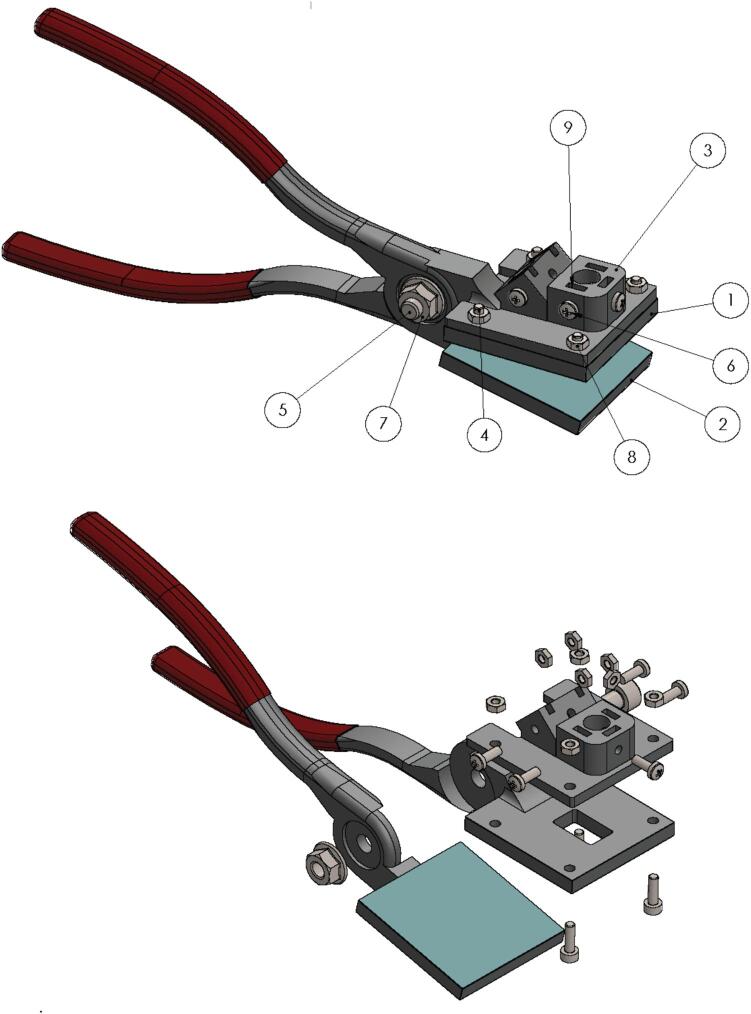
Clamp assembly. Upper: Total assembly. Down: Assembly of the clamp in the exploded view.

**Fig. 3:**
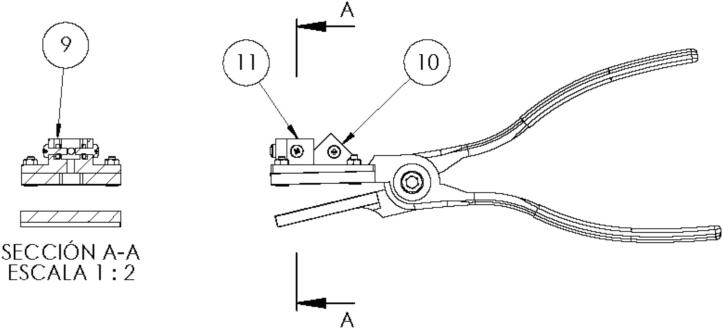
Side view of the gripper.

Fig. 2 shows the assembly of the tool. We used a 3D printer to obtain the clamp and facilitate the construction. For the fabrication of the printed parts, any fused filament fabrication (FFF) or fused granule fabrication (FGF) system can be used [9], [10]. Millimeter bolts and nuts were used for joining the parts, as shown in Fig. 2. The optical cables are connected to the fiber connector support using 5 M3 x 8 mm screws. The fiber optic tips with SMA905 connectors are inserted into the larger diameter holes (10) and (11) in Fig. 3 on the top of the fiber connector support. The tips are then secured by tightening the M3 x 8 mm screws until they are firmly in place.

Table 2 describes each component corresponding to the tool assembly and suggests a construction sequence for the tool. Every step is based on Fig. 2 and Fig. 3.

• First, the files described in Table 1 are 3D printed using PLA (polylactic acid) with a fill density of 20 % on a grid and a layer height of 0.3 mm. All parts are in digital format to be replicated, and a fabrication method can be applied [11]. In addition, the files are available in STL and STEP formats for customization or editing.
• Insert the duckbill plier 1.2 (1) parallel to the duckbill plier 2.2 (2), ensuring the part is well secured. The central holes are the center of components that rotate. To finish the coupling of parts (1) and (2), insert the hexagonal screw m6x16mm (5) in the respective hole and assembly nut m6 (7) with screw m6x16mm and tighten a nut, as shown in Fig. 2.
• Next, place the fiber connector support (3) so that the four holes that place around the base of the workpiece are concentric with the 4 holes of duckbill plier 1.2 (1) to insert the hexagonal screws m3x10mm (4) in the four holes at the near corners of the workpiece and tighten them with the nuts m3 (8).
• Having assembled the above, insert the m3 nuts (8) in the 5 nut holes top (9) available on the fiber connector support (3) as shown in Fig. 3; finally, assemble the m3 x 8 mm screws (6) in perpendicular holes available on the fiber connector support and tighten them. each screw will act as a prisoner.

**Table 1:** Design files.

**Design file name**	**File type**	**Open-source license**	**Location of the file**
Duckbill plier 1.2. step	Step	GNU GPL v3.	https://osf.io/gkdn9/
Duckbill plier 1.2.stl	Stl	GNU GPL v3.	https://osf.io/ru72w/
Duckbill plier 2.2. step	Step	GNU GPL v3.	https://osf.io/rx4tn/
Duckbill plier 2.2.stl	Stl	GNU GPL v3.	https://osf.io/vnw89/
Ensamblaje_plier.step	Step	GNU GPL v3.	https://osf.io/yp7rv/
Fiber connector support.step	Step	GNU GPL v3.	https://osf.io/bmdp4/
Fiber connector support.stl	Stl	GNU GPL v3.	https://osf.io/y8nat/

**Table 2:** Bill of materials.

**Clamp**						
**Designator**	**Component**	**Number**	**Cost per unit −currency**	**Total cost −** **currency**	**Source of materials**	**Material type**
Hexagonal screw m3x10mm	Screw	4	0,12	0,48	https://bit.ly/3A3MK5E	Metal
Hexagonal screw m6x16mm	Screw	1	0,20	0,2	https://bit.ly/3A3MK5E	Metal
Screw m3 x 8 mm	Screw	5	0,12	0,6	https://bit.ly/3A3MK5E	Metal
Nut m6	Screw	1	0,18	0,18	https://bit.ly/3A3MK5E	Metal
Nut m6	Nut	9	0,05	0,45	https://bitg.ly/3dfelYO	Metal
Printing cost	PLA	1	3,25	3,25		Plastic
**Integration with Spectrometer Ocean Insight**						
Spectrometer Flame Miniature spectrometer	Spectrometer	1	5,758	5,758	https://bit.ly/3VPXH66	
Tungsten Halogen Light Sources	Light Sources	1	936	936	https://bit.ly/4arq5zU	
Extreme Solarization Resistant Optical Fibers	Optical Fibers	2	216	432	https://bit.ly/43PIwvM	
Raspberry PI 2	Raspberry PI 2	1	39.97	39.97	https://amzn.to/4arqe6q	

## 6 Operation instructions

**Fig. 4:**
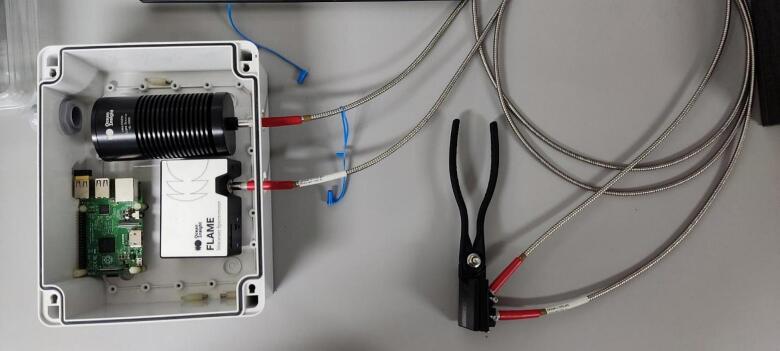
Integration of the clamp with the spectrometer.

After assembling the tool, the clamp can be used with a field and laboratory spectrometer. We use an Ocean Insight Flame S VIS spectrometer [7] to test the clamp for validation. Two optical fibers, SMA905 [12], are used: one for the light source (10) and the other for the acquisition (11). The light source hole is designed to hold the optical fiber at an angle of 45°. Fig. 4 shows the integration of the clamp and the spectrometer.

**Fig. 5:**
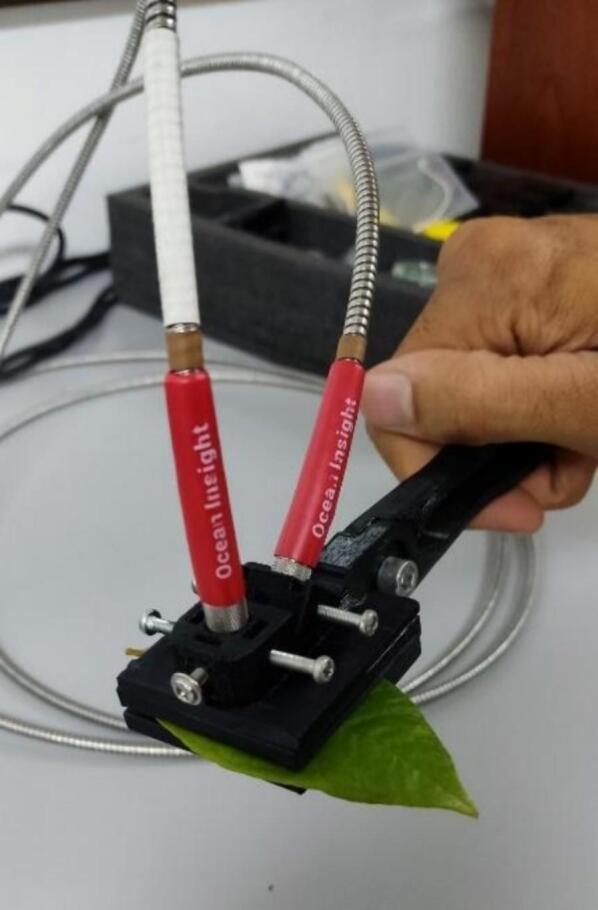
Sampling with the clamp in its completed integration.

After connecting everything, we use the tweezers to grasp any plant leaf to know its spectral signature, as shown in Fig. 5.

The calibration step in spectral signature measurement is of utmost importance. It involves the collection of white and black signatures from reflectance patterns. This step can be done before and after a collection session. The reflectance of each leaf is obtained using equation (1), where λ is the wavelength (nm), r the measured radiance from the leaf (W m^-2^sr^-1^: watts per square meter and steradian), $r_{\mathrm{black}}$ the radiance from the black pattern (W m^-2^sr^-1^), and $r_{\mathrm{white}}$ the radiance from the white pattern (W m^-2^sr^-1^). Also, it is recommended to collect at least 10 signatures from the same leaf and obtain their average to reduce noise effects. See (Table 3).

(1) $$R\left(\lambda \right)=\frac{r\left(\lambda \right)-r_{\mathrm{black}}\left(\lambda \right)}{r_{\mathrm{white}}\left(\lambda \right)-r_{\mathrm{black}}\left(\lambda \right)}$$

**Table 3:** Assembly parts list.

Part number	Name of the part	Quantity
1	Duckbill plier 1.2	1
2	Duckbill plier 2.2	1
3	Fiber connector support	1
4	Hexagonal screw m3 x 10 mm	4
5	Hexagonal screw m6 x 16 mm	1
6	Screw m3 x 8 mm	5
7	Nut m6	1
8	Nut m3	9
9	Nut hole	1
10	Light source hole	1
11	Spectrometer fiber hole	1

## 7 Validation and characterization

We use two maize crops in controlled conditions for testing and validation at La Selva Research Center (AGROSAVIA, Rionegro, Colombia). We use a FLAME S VIR NIR spectrometer from Ocean Insight integrated with a 2-meter QP600-2-VIR-NIR optical fiber. The spectrometer range is between 350 and 1000 nm, capturing 2049 bands. The signatures are captured using two protocols. First, we collect the spectral signature over the leaf, positioning the probe fiber at a 2 cm distance. In this first setup, we use sunlight as the source. The second setup used the designed clamp. In this case, we employed an HL-2000-LL light source from Ocean Insight. The light source has a wavelength range from 360 to 2400 nm, with a tungsten halogen bulb and a light output stability of 0.25 % peak-to-peak. The intensity (counts per ms of exposure time) is upper 1.E + 06 between 400 to 900 nm. Several tests were performed to determine the optimum clamp design. The captured spectrum results from an average of 10 measurements to improve the signal-to-noise ratio. Black and white calibration spectra were collected before the measurements on the leaves.

**Fig. 6:**
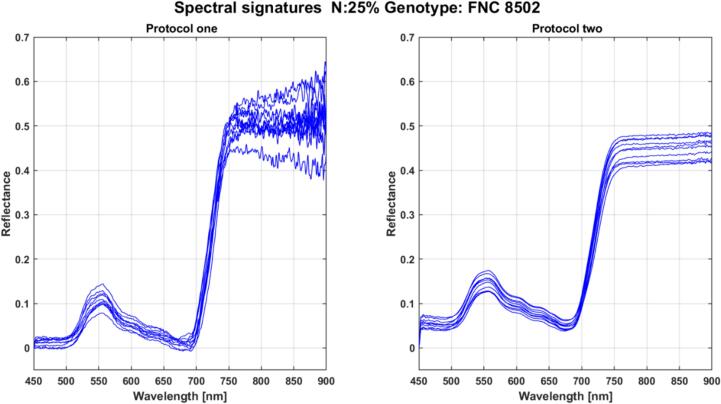
Spectral signatures captured in maize leaves (genotype FNC 8502) subjected to nitrogen nutrient deficiency. Left: signatures captured using sunlight. On the Right: signatures captured using a light source and the designed clamp.

Fig. 6 (left side) presents spectral signatures collected over maize leaves using the first protocol after calibration using the black and white references. The obtained spectrum has high noise levels between 400 and 450 nm and 840 nm to 1000 nm. Noise in the near-infrared region is due to changes in illumination, leaf movement, as well as the intrinsic spectral variability of plants. This situation was the main inspiration for designing a clamp that uses the spectrometer in the field and laboratory at a low cost. Fig. 6 (right side) shows the spectral signatures captured using our clamp and the light source. In this case, we can note an improvement in the signal-to-noise ratio. This is due to two factors: the designed clamps allow a light source, removing the perturbation caused by sunlight and the atmosphere. Second, it fixes the leaf, eliminating possible disturbances caused by the wind. The signature captured with the clamp maintains the spectral variability of the plant, reducing the effect of external factors. A complete spectral library collected with the proposed system can be found in [16]. Note that sunlight protocols for spectra measurement are standard in several spectrometry applications, such as spectral albedo measurements [18]. A system like the one used in [18] requires more robust equipment (we used a low-cost spectrometer) and a more robust calibration model. Thinking about reducing both the cost and the complexity of obtaining a reliable vegetation measurement, the device presented in this article seeks to work with low-cost equipment, reducing effects due to changes in lighting and for the movement of leaves while facilitating the calibration process.

## 8 Ethics statements

This work did not involve human subjects or animal experiments.

## CRediT authorship contribution statement

**Camilo Acevedo-Correa:** Conceptualization, Investigation, Methodology, Validation, Writing – original draft. **Manuel Goez:** Conceptualization, Investigation, Methodology, Validation. **Maria C. Torres-Madronero:** Conceptualization, Funding acquisition, Investigation, Methodology, Project administration, Writing – review & editing. **Tatiana Rondon:** Conceptualization, Writing – review & editing, Investigation, Validation.

## Declaration of competing interest

The authors declare that they have no known competing financial interests or personal relationships that could have appeared to influence the work reported in this paper.
